# *In-Silico* Prediction of Key Metabolic Differences between Two Non-Small Cell Lung Cancer Subtypes

**DOI:** 10.1371/journal.pone.0103998

**Published:** 2014-08-05

**Authors:** Alberto Rezola, Jon Pey, Ángel Rubio, Francisco J. Planes

**Affiliations:** Department of Bioinformatics, CEIT and TECNUN, University of Navarra, San Sebastián, Spain; University of Nebraska Medical Center, United States of America

## Abstract

Metabolism expresses the phenotype of living cells and understanding it is crucial for different applications in biotechnology and health. With the increasing availability of metabolomic, proteomic and, to a larger extent, transcriptomic data, the elucidation of specific metabolic properties in different scenarios and cell types is a key topic in systems biology. Despite the potential of the elementary flux mode (EFM) concept for this purpose, its use has been limited so far, mainly because their computation has been infeasible for genome-scale metabolic networks. In a recent work, we determined a subset of EFMs in human metabolism and proposed a new protocol to integrate gene expression data, spotting key 'characteristic EFMs' in different scenarios. Our approach was successfully applied to identify metabolic differences among several human healthy tissues. In this article, we evaluated the performance of our approach in clinically interesting situation. In particular, we identified key EFMs and metabolites in adenocarcinoma and squamous-cell carcinoma subtypes of non-small cell lung cancers. Results are consistent with previous knowledge of these major subtypes of lung cancer in the medical literature. Therefore, this work constitutes the starting point to establish a new methodology that could lead to distinguish key metabolic processes among different clinical outcomes.

## Introduction

**Lung cancer** is the most common cancer worldwide both in terms of cases and deaths and its highest incidence rates belong to Europe and North America [1]. With the advent of -omics data, much effort has been made to identify mutations and oncogenes in different lung cancer subtypes, aiming to develop more effective treatments. However, prognosis is still poor and further research is required to elucidate novel biomarkers and treatments that improve clinical outcomes [2].

In this context, the study of **metabolic processes** in cancer is currently a hot topic, as we have an increasing evidence of its re-programming. Apart from glucose metabolism, the so-called Warburg effect, alterations have been reported in the synthesis of nucleotides, amino acids and lipids [3], as well as relevant mutations in metabolic genes and accumulations of key metabolites [4]. As tumor cells exhibit high genetic diversity, the identification of relevant metabolic pathways in different cancer sub-types represents an important research area.

**High-throughput -omics** technologies have brought about a novel scenario where a more complete analysis of metabolism is possible. A major advance was the reconstruction of the human genome-scale metabolic network [5], [6], which allowed researchers to analyze human metabolism in different scenarios at an unprecedented level of complexity, using theoretical methods and -omics data [7], [8]. In this direction, different **network-based metabolic pathway concepts** have been introduced in the last years [9]. They have shown that cellular metabolism involves a more complex and varied pathway structure than those presented in canonical maps. In particular, a promising concept is that of **Elementary Flux Modes** (EFMs), which allows us to decompose a metabolic network into its simplest modes of behaviour [10]. However, the integration of -omics data with EFMs to analyze human metabolism has been limited, due to the fact that the computation of EFMs is hard in genome-scale networks. This issue has been recently addressed in [11], where a new protocol to integrate gene expression data and EFMs is proposed. This approach was successfully applied to identify metabolic differences among several healthy tissues.

Based on [11], our objective here is to identify key metabolic pathways and metabolites in two major subtypes of non-small cell lung cancer (NSCLC): adenocarcinoma and squamous-cell carcinoma. In particular, we aim to investigate if specific differences between these subtypes can be found combining EFMs and gene expression data. According to previous knowledge of these major subtypes of lung cancer in the medical literature, our results properly distinguish key metabolic processes among the different clinical outcomes analyzed.

## Materials and Methods

### Elementary Flux Modes (EFMs) concept

**Figure 1:**
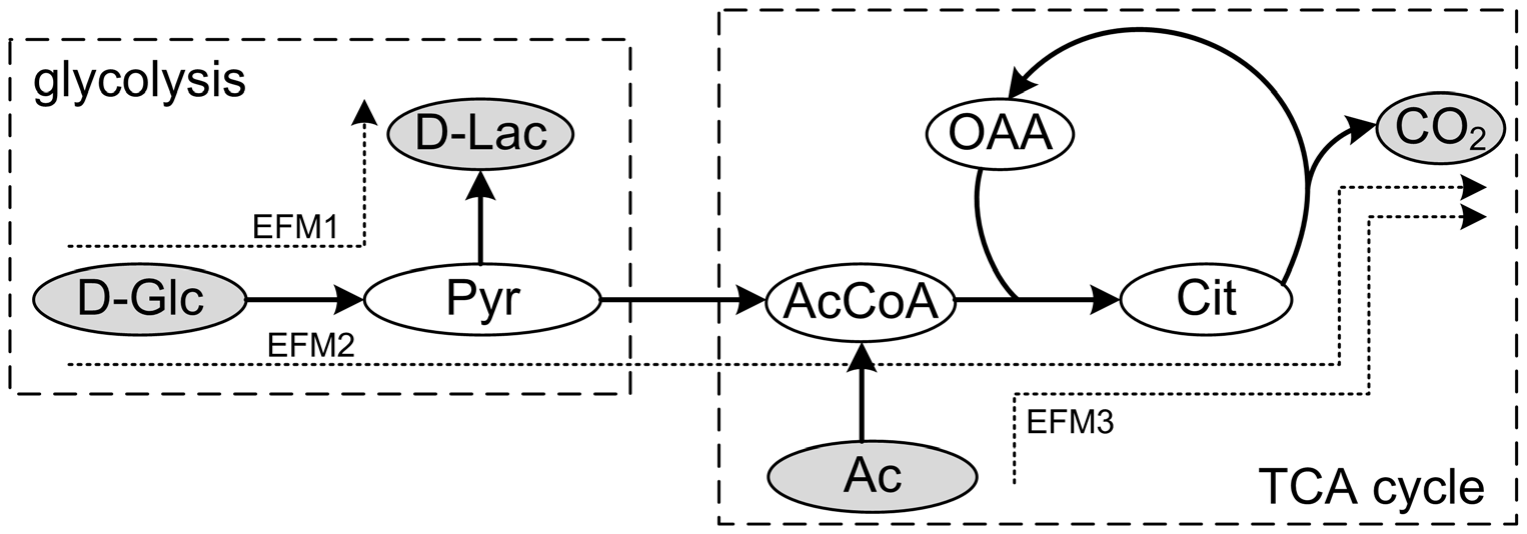
Example network that represents a simplified system involving glycolysis and TCA cycle. Abbreviations: Ac, Acetate; AcCoA, Acetyl-CoA; Cit, Citrate; D-Lac, lactate; D-Glc, glucose; OAA, oxaloacetate, Pyr, Pyruvate, CO2, Carbon dioxide.

To illustrate the concept of EFMs, we used Figure 1, which represents a simplified metabolic system involving glycolysis and TCA.

An EFM is technically a minimal subset of enzymes able to perform in sustained steady-state. Steady-state implies that metabolites inside the boundaries of the system, e.g. *pyruvate* (Pyr), must be in stoichiometric balance, i.e. flow in must be equal to flow out. This condition requires the definition of metabolites able to be exchanged outside the system, namely here the inputs are *glucose* (D-Glc) and *acetate* (Ac), while the outputs *lactate* (D-Lac) and *carbon dioxide* (CO2). In addition, “minimal” means that the removal of an enzyme leads to pathway disruption. In our example in Figure 1, we have 3 EFMs. EFM1 represents anaerobic glycolysis; EFM2 aerobic glycolysis via TCA cycle; EFM3 TCA cycle fed by acetate. It is easy to check that they satisfy the conditions mentioned above. For more technical details, please see [10].

Note that EFMs are minimal modes of behaviour and combinations are also possible. However, in different scenarios some of them may prevail over the others. For example, cancer cells produce energy primarily via anaerobic glycolysis (EFM1) even when sufficient *oxygen* is available (Warburg effect). Note that EFMs typically have different inputs (substrates) and outputs (excreted metabolites). In this article, we aim to exploit this idea to separate different clinical scenarios based on gene expression data.

### Human EFMs collection and lung cancer data

Here we used a subset of 5875 EFMs previously determined in [11] from Recon 1 human metabolic network [5], which contains 2469 biochemical reactions and 1587 metabolites. This subset of EFMs involves a diverse list of metabolic pathways potentially active in different human physiological conditions (see [11] for more detailed information of this set of EFMs).

On the other hand, gene expression data was extracted from Gene Expression Omnibus (GEO) database [12]. In particular, we considered 58 human NSCLC tumor tissue samples from [13], and 6 normal lung tissue samples (CN) from [14]. 40 NSCLC tumor tissue samples were taken from patients clinically diagnosed as adenocarcinoma (AD), while the other 18 tumor tissue samples from patients with squamous-cell carcinoma (SQ). All these samples were hybridized in an Affymetrix array HGU 133 plus, which contains 54.675 probes for 20.283 genes. We describe below different methods applied to analyze this data.

### Differential expression analysis

We determined which genes were over-expressed, unchanged or under-expressed in AD with respect to SQ (upAD) and vice versa (upSQ). Note that over-expressed genes in AD are down-expressed in SQ, and vice versa, as can be observed in the first column of Table 1. In addition, gene expression data from healthy tissues cannot be directly compared with data from cancer tissues as they belong to a different data source. This does not constitute an issue, as we are focused on elucidating differences between AD and SQ.

**Table 1:** Resulting gene/reaction expression and characteristic EFMs.

Scenario	Genes (1/0/-1)	Reactions (1/0/-1)	Characteristic EFMs
AD	1096/9265/9922	181/887/421	165
SQ	1317/8145/10821	240/719/530	207
CN	1353/5131/13799	262/494/733	228
upAD	1725/16163/2395	333/1017/139	116
upSQ	2395/16163/1725	267/985/237	267

Abbreviations stand for adenocarcinoma (AD), squamous-cell carcinoma (SQ), control (CN), up-regulated genes in AD with respect to SQ (upAD) and up-regulated genes in SQ with respect to AD (upSQ). Up-regulated/highly-expressed, unchanged/normally-expressed and down-regulated/lowly-expressed gene/reactions are denoted 1, 0 and -1, respectively.

For this task we used limma package in R statistical software [15], i.e. multiple linear regressions and empirical bayes statistics that determine the likelihood of a gene not being differentially expressed between both conditions (*p*-value). Then, false discovery rate (FDR) technique was applied to correct the effect of multiple hypotheses testing, transforming previous *p*-values into *q*-values [16]. We considered differentially expressed genes those with a *q*-value lower than 5%. Note that this threshold is arbitrary, however, the smaller the threshold, the greater the confidence level. The determination of up- and down-regulated genes is straightforward based on linear regression coefficients.

### Absolute expression analysis

We define absolute expression analysis as the classification of genes as present or absent in a specific biological sample. Here we particularly classified genes into three states: highly-, normally- and lowly-expressed. This discrete classification of genes was made for every group: AD, SQ and CN. Note that this analysis was conducted for each group separately and no comparison between groups was made, i.e. absolute expression of genes is a functional property for each group.

For this purpose we first classified genes in each sample as active or inactive based on the Gene Expression Barcode model [17]. Then, in order to obtain the desired three level classification, we defined a gene as highly (lowly) expressed if all the probes containing such gene are active (inactive) in the entire set of samples, and moderately expressed otherwise. A less stringent threshold (for example 95% of the probes instead the 100%) can be selected, but the confidence level of gene high- and low-expression will be decreased.

### Data transformation: from genes to reactions

Differential and absolute gene expression leads to an up-regulated/highly-expressed, unchanged/normally-expressed and down-regulated/lowly-expressed gene classification.

In order to map this gene expression classification into the set of metabolic reactions included in the set of EFMs selected, we used the Boolean laws, also known as Gene-Protein-Reaction (GPR) rules, reported in [5], as previously done in [11], [18]. Note here that Recon 1 human metabolic network reconstruction annotates 1496 genes from which 1451 are found in the HGU 133 plus array.

Based on these GPR rules and the gene expression categorization, we obtain a three level metabolic reaction classification, i.e. up-regulated/highly-expressed, unchanged/normally-expressed and down-regulated/lowly-expressed reactions.

### Characteristic and differential EFMs

We mapped the reactions classification into the set of 5876 EFMs in each scenario: AD, SQ, CN, upAD and upSQ. For AD, SQ and CN scenarios, we determined a subset of characteristic EFMs with the approach presented in [11]. We define **characteristic EFMs** as those significantly enriched with highly expressed reactions and involving a small number of lowly expressed reactions. This definition focuses on absolute expression data (see previous “Absolute expression analysis” subsection).

This concept can be extended for differential expression data, in our case using the gene sets obtained for upAD and upSQ scenarios. In particular, we define **differential EFMs** as those significantly enriched with over-expressed reactions and involving a small number of under-expressed reactions. Note that the use of differential expression involves several theoretical issues, e.g. genes consistently highly expressed may have a small fold change.

As the combination of differential EFMs with characteristic EFMs is a more accurate approach, in this paper we coined the term **“prominent” EFMs** for those EFMs that are characteristic and differential at the same time, i.e. they are significant both in the absolute and the differential analyses.

## Results and Discussion

Around 70% of diagnosed lung cancers are Non Small Cell Lung Cancers (NSCLC) and belong to two main subtypes, particularly AD and SQ. The objective of this work is to elucidate specific metabolic properties and differences between AD and SQ lung cancers. To that end, the methodology presented above was used.

Table 1 summarizes the results for the absolute and differential expression analysis accomplished. Interestingly, in Table 1 we found that the number of lowly expressed genes and reactions in CN is significantly higher than in two cancer scenarios. This may suggest that the reprogramming of cancer metabolism enhances systemic robustness, probably activating silenced pathways that guarantee and optimize proliferation. Note however that, to a much lesser extent, the number of highly expressed reactions is higher in CN. These insights may indicate that metabolism in CN is more specific than in cancer and presents less variability across samples in the active set of enzymes. The same conclusion is achieved with moderately expressed genes. On the other hand, if we focus on the differential analysis, we found, as expected by construction, that up-regulated genes in upAD are down-regulated in upSQ, and vice versa, e.g. the subset of 1725 up-regulated genes in AD (1 in upAD) matches the subset of down-regulated genes in SQ (-1 in upSQ). However, the same does not occur at the reactions level due to GPR rules, which illustrates the regulatory complexity of metabolism.

**Figure 2:**
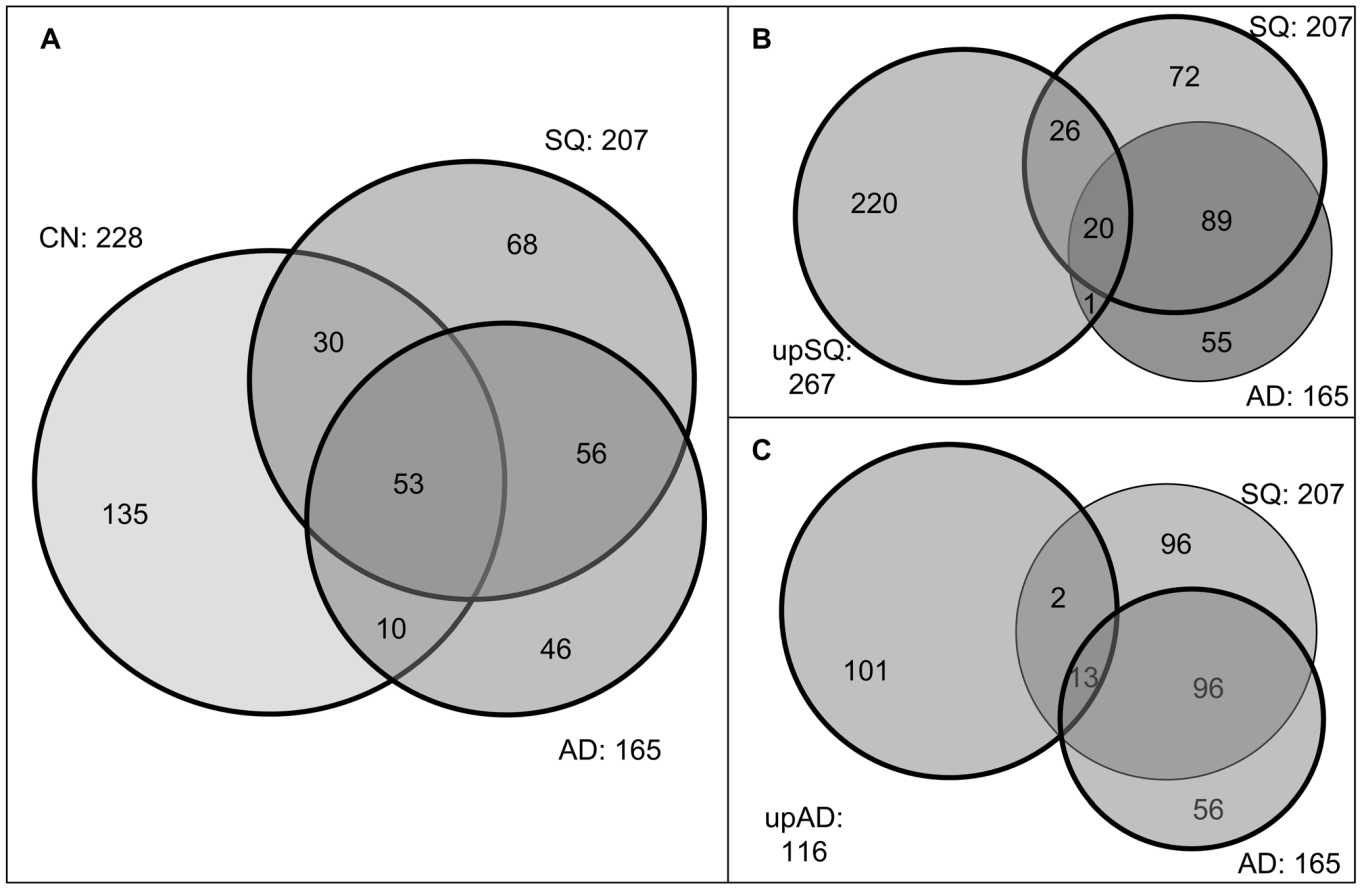
Venn diagrams proportional to the number of characteristic EFMs in: A) AD, SQ & CN; B) SQ, upSQ & AD; C) AD, upAD & SQ.

We selected as **differential** and **characteristic EFMs** for each scenario those with a FDR lower than 20%. The total number of statistically significant EFMs can be found in Table 1. In particular, we found a considerable number of characteristic EFMs in each condition and, as discussed in [11], their number was not necessarily proportional to the number of highly- and lowly-expressed (up- and down-regulated) reactions. Therefore, it is not surprising that more characteristic EFMs are found in SQ than in AD, since the number of characteristic and differential EFMs depends on the connectivity of highly- and lowly-expressed (up- and down-regulated) reactions in our set of EFMs [11]. In order to visualize and interpret differential and characteristic EFMs, we mapped them into the Venn diagrams shown in Figure 2.

Figure 2.A shows the number of characteristic EFMs that overlap in CN, SQ and AD. As partially expected, it can be observed that the metabolic activity in cancer tissues (AD and SQ) is more similar than in healthy tissue (CN). In particular, a common subset of 109 characteristic EFMs is found for AD and SQ. Among them, 56 are not involved in CN, which may represent a core metabolic network of lung cancer metabolism. The other 53 EFMs are common for our three scenarios, revealing similarities among cancer and healthy tissues.

In order to detect more specific pathways and metabolites in AD and SQ, we compared characteristic and differential EFMs of SQ and AD, as shown in Figures 2.B and 2.C, respectively. As noted above, we considered as SQ prominent those EFMs characteristic in SQ and up-regulated in SQ; analogously, AD prominent EFMs are characteristic in AD and up-regulated in AD. We identified **46 SQ prominent EFMs** and **13 AD prominent EFMs**, i.e. characteristic EFMs which are at the same time differential EFMs. However, from these EFMs, 20 SQ prominent EFMs and 13 AD prominent EFMs were found to be characteristic in both cancer tissues. This implies that, though being present in both tissues, their activity is higher in SQ and AD, respectively. In addition, we detected one clear false positive in SQ differential EFMs, i.e. an EFM differentially expressed in SQ which is only characteristic in AD, and two false positives in AD differential EFMs.

### AD and SQ prominent input/output metabolites

**Figure 3:**
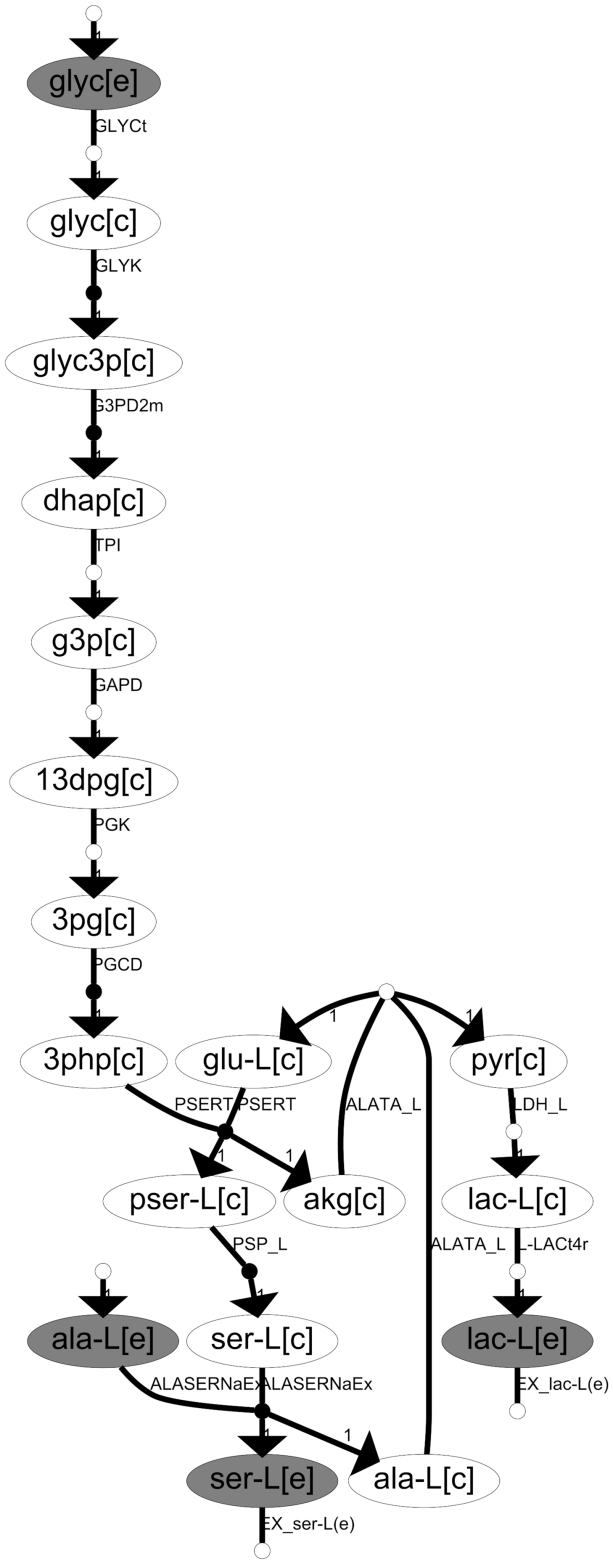
Illustration of an SQ prominent EFM and its input and output metabolites. Ellipses represent metabolites and arrows represent reactions. White and black dots within the arrows represent reversible and irreversible reactions, respectively. Each metabolite is depicted with its corresponding compartment shown in brackets: [e], extracellular, and [c], cytosol. Grey and white ellipses represent external and internal metabolites, respectively. Nomenclature for metabolites and reactions was taken from [5] and it is included in File S1.

The results above show that our approach is able to distinguish **prominent EFMs** in AD and SQ lung cancers. In order to translate this information into more practical insights, we focused on input and output metabolites involved in these 46 SQ and 13 AD prominent EFMs, respectively. For illustration, consider Figure 3, which shows an SQ prominent EFM consuming *glycerol* (glyc) and *L-alanine* (ala-L), input metabolites, and producing *L-serine* (ser-L) and *L-lactate* (lac-L), output metabolites.

As these EFMs were obtained from a human genome-scale metabolic network, these input and output metabolites mainly correspond to substrates (uptake) and excreted products in lung cancer and, therefore, could be measured in bio-fluids and be spotted as biomarkers. Hence, this approach could complement metabolomic studies.

We here analyzed input/output metabolites involved in AD and SQ prominent EFMs. In particular, we identified input/output metabolites present in the 46 SQ and upSQ EFMs, but not in upAD and if possible (not in) AD and CN. These metabolites are termed SQ prominent. We hypothesize for SQ prominent metabolites a higher uptake (inputs) and secretion (outputs) flux than in AD and, therefore, they could be used to distinguish SQ and AD. The same can be done for the 13 AD and upAD EFMs. However, no results of interest were found in this case.

Table 2 shows a summary of the most relevant SQ prominent uptake and secretion metabolites obtained. Full details can be found in File S1. Based on literature, we discuss below previous works as to the role of these metabolites.

**Table 2:** SQ prominent secretion and uptake metabolites.

Names	CN	AD	up AD	SQ	upSQ
*S_1_*: *Deaminoneuraminic acid*	1	-	-	5	5
*S_2_*: *Methylglyoxal*	2	-	-	4	4
*S_3_*: *Tetrahydrofolate*	-	-	-	1	1
*S_4_*: *Acetone*	-	-	-	1	1
*S_5_*: *alpha-N-Phenylacetyl-L-glutamine*	-	-	-	1	1
*U_1_*: *Acetoacetate*	-	4	-	5	5
*U_2_*: *D-Mannose*	2	-	-	4	4
*U_3_*: *Glycerol*	1	1	-	4	4
*U_4_*: *Heptaglutamyl folate*	-	-	-	1	1
*U_5_*: *L-Phenylalanine*	-	-	-	1	1
*U_6_*: *L- Glutamate*	-	-	-	1	1

Entries in the table count for the number of characteristic and differential EFMs involving a particular uptake U or secretion S metabolite.

A higher intracellular abundance *methylglyoxal* in SQ in comparison with AD has been previously reported in [19], which constitutes a clear success of our approach. In addition, a high expression of *deaminoneuraminic aci*d in both SQ and AD tissues was found in [20]. However, our approach predicted a more relevant role in SQ, which requires further research to be validated. Note also that both *methylglyoxal* and *deaminoneuraminic aci*d are typically incorporated into different proteins and their presence in different biofluids has not been explored.

Given the results presented in [19], one may question the reason as to why *deaminoneuraminic acid* is not involved in the subset of characteristic EFMs in AD. It must be observed that our source of evidence is gene expression data. In SQ we found a regular expression pattern for genes involved in EFMs producing *deaminoneuraminic acid*, but not in AD. This does not exclude the importance of *deaminoneuraminic acid* in AD, as post-transcriptional changes may occur.

With respect to *tetrahydrofolate* and *heptaglutamyl folate*, a different performance of *folate* metabolism in AD and SQ has been recently elucidated [21]. In that work, it is reported that *gamma-glutamyl hydrolase* enzyme, which removes polyglutamate chains from polyglutamylated folate, facilitating escape of folate from within the cells, is higher in SQ than AD. This is particularly in line with our hypothesis.

In [22], they found a higher but non-significant level of *L-Phenylalanine*, *glutamate* and *glycerol* in serum from AD patients. This is in consonance with our results, which suggest a major cellular uptake of these metabolites in SQ. For other amino acids appearing in our set of EFMs (*L-alanine*, *glutamine* and *L-serine*), not shown in Table 2, the comparison between AD and SQ is not available in [22]. However, they found a significant alteration of their serum levels between lung cancer and healthy patients.

For the rest of the metabolites, further research is required to validate their function in AD and SQ. However, aside from *D-mannose*, we could find clear association of these metabolites with cancer. For example, in [23], a higher release of *acetone* was found in breath of lung cancer patients than in healthy volunteers. In addition, a lower level of *acetoacetate* was found in malignant pleural effusions [24]. On the other hand, the accumulation of *alpha-N-Phenylacetyl-L-glutamine* has been previously hypothesized as an urinary biomarker for bladder cancer [25] and, therefore, it constitutes an attractive hypothesis to be explored.

Note here that, as shown in Figure 1, EFMs may combine different canonical metabolic pathways and pieces of them. In our set of 46 SQ prominent EFMs, the most frequent canonical pathways are the degradation of *glycerol* and *D-mannose*, as well as the biosynthesis of *serine* and *glutamine*.

Given the results provided in Table 2, it is clear that EFMs are more informative than canonical pathways, as the list of potential metabolites is wider when EFMs are directly interrogated. This shows the potential of our approach with respect methods based on canonical pathways.

## Conclusions

The concept of Elementary Flux Mode is not new in Systems Biology [10]. However, their computation has been not possible in genome-scale networks until recently, which has restricted their use to analyze -omics data. With the advent of optimization-based techniques [26]–[29], the performance of algorithms to compute EFMs in genome-scale metabolic networks is rapidly improving. These advances have allowed us to determine a significant set of EFMs in different organisms, as shown in [11] for human metabolism. Based on them and -omics data, a more accurate picture of metabolic processes will be obtained in different scenarios.

In this article we have identified key EFMs in both AD and SQ NSCLC based on gene expression data, finding a different metabolic signature. To that end, we used and extended the approach presented in [11], with a gene classification based on i) absolute and ii) differential expression analysis, which are complementary and allows us to have more accurate results.

There has been much debate in the literature about the correlation between mRNA and protein levels, as well as metabolic fluxes. The extent to which transcriptomic data correlates with proteomic and fluxomic data is still an open question [30]. However, different recent articles have shown the relevance of gene expression data for predicting metabolic phenotypes, e.g. [7], [31], which illustrates the value of approaches as the one presented here. Note also that our approach is general and could be applied to proteomic and metabolomic datasets, overcoming post-transcriptional changes.

Our approach was used to identify input and output metabolites with a different activity in AD and SQ NSCLC. We found a number of these metabolites, finding a good agreement with previously reported literature. Our approach, based on EFMs and gene expression data, open new avenues for studying novel biomarkers (metabolites) characterizing different clinical outcomes. Note that the amount of gene expression data in different cancer cell lines and patients is massive [12]. Using this data in the context of the approach presented here could guide metabolomic experiments and biomarker discovery in plasma/urine samples, particularly given the difficulty of interpreting metabolomics spectra in non-targeted approaches.

## Supporting Information

File S1 Contains four excel worksheets defining i) the names and abbreviations of the reactions and metabolites involved in the used human metabolic network reconstruction [5], ii) details of EFMs selected as characteristic/differential from a general set compiled in [11], iii) activity of EFMs selected as characteristic/differential in different lung cancer scenarios, and iv) an extension of Table 2, including full details of SQ and AD specific uptake and secreted metabolites.
(XLSX)
